# Naringinase Biosynthesis by *Aspergillus niger* on an Optimized Medium Containing Red Grapefruit Albedo

**DOI:** 10.3390/molecules27248763

**Published:** 2022-12-10

**Authors:** Joanna Bodakowska-Boczniewicz, Zbigniew Garncarek

**Affiliations:** Department of Biotechnology and Food Analysis, Wroclaw University of Economics and Business, 53-345 Wroclaw, Poland

**Keywords:** naringinase, biosynthesis of enzymes, grapefruit albedo, *Aspergillus niger*

## Abstract

This study aimed to develop a method of naringinase biosynthesis by *Aspergillus niger* KMS on an optimized culture medium. The concentration of the six medium components in shake flasks was optimized by the Box and Wilson factor gradient method. Naringinase’s substrate, naringin, powdered albedo, flavedo, and red grapefruit segment membranes were used to stimulate naringinase biosynthesis. Rhamnose was chosen as the carbon source, while the nitrogen source was yeast extract and sodium nitrate. Naringinase biosynthesis was most favorable in the culture medium with the following composition (g 100 mL): 3.332—NaNO_3_; 3.427—yeast extract; 0.184—KH_2_PO_4_; 0.855—red grapefruit albedo; 0.168—naringin; 2.789—rhamnose. The obtained *Aspergillus niger* KMS culture fluid was concentrated, thereby precipitating the protein. As a result, a naringinase preparation with high activity, equal to 816 µmol × min^−1^ × g^−1^, was obtained.

## 1. Introduction

Naringinase is an enzyme with the dual activity of α-L-rhamnosidase (EC 3.2.1.40) and β-D-glucosidase (EC 3.2.1.21). Naringinase is used in the deglycosylation of compounds with high application potential in the food and pharmaceutical industries [1,2,3]. Naringinase is primarily significant in processing citrus fruits, reducing their bitter taste [4]. Naringin, a flavonoid glycoside, is mainly responsible for the bitter taste of citrus fruits [4,5,6,7]. Naringin is present in grapefruits, giving them their characteristic bitter taste [8]. One of the naringinase subunits, α-L-rhamnosidase, hydrolyzes naringin to rhamnose and prunine; β-D-glucosidase is hydrolyzed, then prunine is converted to glucose and unflavored naringenin.

Ferreira et al. [9] observed a reduction of naringin concentration due to its hydrolysis by naringinase, which improves the commercial value of citrus juices and retains their health-promoting properties at the same time [8]. Initially, naringinase was isolated from plant sources, including celery seeds and grapefruit leaves. Various microorganisms can produce naringinase, including species from the genus *Penicillium* [10,11,12,13,14], *Rhizopus* [15], *Staphylococcus* [16], *Streptomyces* [17], and *Bacillus* [18]; however, the most significant interest of the authors is focused on strains of the genus *Aspergillus* [18,19,20,21,22,23,24,25,26]. Many molds of the genus *Aspergillus* can synthesize naringinase: *A. niger* [19,22,23,27,28,29,30,31,32,33,34,35], *A. oryzae* [18,20,33], *A. aculetaus* [24,36], *A. brasiliensis* [37], *A. sojae* [21], *A. terreus* [38], *A. foetidus* [33], *A. flavus* [39,40,41], and *A. tubingensis* [42].

Most often, the authors researched naringinase biosynthesis in submerged cultivation of *A. niger* strains [19,22,23,27,29,30,31,43]. *A. niger* is a microorganism classified as GRAS (Generally Recognized As Safe). According to the Food and Drug Administration (FDA), it also belongs to the category of microorganisms safe for food and medical uses. It is essential due to the potential use of naringinase preparation in the food industry. *A. niger* is also used to produce other enzymes, food additives, and traditional fermented foods (fermented Chinese soybean paste) [23]. The efficiency of naringinase biosynthesis can be easily increased by optimizing the culture parameters [29,44]. For these reasons, *A. niger* species can be considered the most important source in naringinase preparation. The biosynthesis of naringinase by *A. niger* is influenced by many factors, e.g., the strain molds, the culture medium’s composition, and the process’s conditions. One of the most important factors influencing the culture of *Aspergillus* molds is the composition of the substrate. The presence of an inducer, carbon source, nitrogen source, and the content of mineral salts are the main factors determining the growth and activity of microorganisms [15,45]. Naringinase is an inducible enzyme [34,46] and the continuous or gradual addition of an inducer increases the production of this enzyme [19,27]. Different substances can stimulate the biosynthesis of naringinase, e.g., naringin, naringenin, routine, hesperidin, and rhamnose. [11,12,15,18,19,21,23,27,29,31,32,34,39,41,43,47,48,49]. 

In recent years, natural particles of citrus fruit have been used as a rich source of naringin and thus as inducers of naringinase biosynthesis [20,33,44,50]. It should be noted that few authors have conducted studies on naringinase biosynthesis using citrus albedo as a component of the culture medium. Ye [51] described a method of naringinase production by submerged cultivation of *A. niger* on a medium containing dried orange peel powder. Furthermore, Borkar et al. [44] obtained naringinase using an *A. niger* van Tieghem MTCC 2425 submerged culture on a medium containing orange solid waste. Chen et al. [20] worked on the naringinase biosynthesis by *A. oryzae* JMU316, and, as the only carbon source, they used pomelo peel powder. Puri et al. [52] obtained bacterial naringinase from *Staphylococcus xylosus* MAK2; for this purpose, they used dried waste from orange processing as a culture medium component. In order to produce naringinase, molds of the *Aspergillus* genus (including *A. foetidus*, *A. niger*, and *A. niger* HPD-2) were also cultured on a solid medium containing orange and grapefruit peels [33]. Naringinase was also obtained from the cultures of *A. niger*, *P. nalgiovense*, *A. flavus*, and *A. terreus* on a solid medium containing orange peels [53]. The type and concentration of carbon and nitrogen sources in the culture medium play a significant role in naringinase biosynthesis. The authors mainly used saccharides as a carbon source for the growth of the molds and the production of naringinase, e.g., rhamnose [22,29,32,34,43], glucose [29,40,41,43], maltose [18,29,37], sucrose [29], fructose [29], and starch [21,29]. Natural raw materials, by-products of processing processes, are also used as a carbon source. The authors also use the above-mentioned dried albedo and pomelo peel as carbon sources, which is a waste by-product from processing fruit juices. They are readily available and inexpensive compared to other compounds, which makes them a good carbon source [20]. In 2012, Ye et al. [51] patented a method of obtaining naringinase during the cultivation of *A. niger* in a medium containing powder from dried orange peels as a carbon source. It can be assumed that other citrus peels can also be used as a carbon source for naringinase production. Puri et al. [52], for the biosynthesis of bacterial naringinase—apart from natural citrus particles—used an additional carbon source in the form of sucrose. Furthermore, Borkar [44], in addition to orange particles, in the composition of the medium included an additional carbon source—rhamnose. In the experiments conducted using citrus albedo, the remaining researchers did not add compounds to the culture medium that were an additional carbon source or a naringinase biosynthesis stimulator. Using dried citrus fruit peels as components of the culture medium is an alternative way of managing waste from citrus fruit processing. The amount of waste produced by the fruit processing industry increases every year. It is reported that in China, waste in the form of pomelo peels only amounts to about 100,000 tons per year [20]. Citrus peels can be used to extract essential oils or naringin [35,52], but the costs of these applications are high [20]. The biosynthesis of enzymes by microorganisms promotes the utilization of agricultural waste, an excellent source of carbon or nitrogen for microorganisms in the production of biocatalysts [40]. This study aimed to develop a method of naringinase biosynthesis by *A. niger* KMS on a medium containing natural stimulants for this enzyme’s production, which were derived from waste or by-products from grapefruit juice—i.e., albedo skins and citrus seed nests.

The goal of this work required determining the influence of temperature and optimizing the culture medium composition to maximize naringinase production.

## 2. Results and Discussion

### 2.1. Optimization of A. niger KMS Submerged Cultures Run in Shaker Flasks

#### 2.1.1. Selection of the Carbon Source in the Culture Medium 

**Figure 1:**
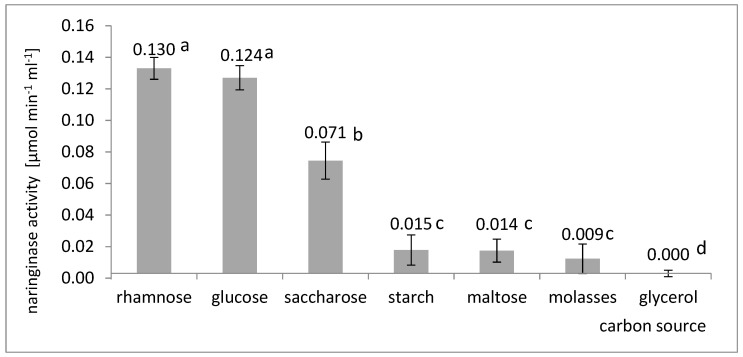
Influence of the type of carbon source on the last activity of naringinase in the post-culture fluid in submerged batch cultures with *A. niger* KMS carried out in flasks on a shaker on the Czapek medium with the addition of naringin. Various letter markings indicate statistically significant differences at *p* < 0.05.

Naringinase activity was compared in the culture of *A. niger* KMS on a medium containing rhamnose, glucose, sucrose, starch, maltose, glycerol, or molasses (Figure 1).

The data analysis presented in Figure 1 shows that *A. niger* KMS prefers to use rhamnose as a carbon source for the biosynthesis of naringinase. The highest activity of naringinase, equal to 0.130 µmol × min^−1^ × mL^−1^, was obtained on the culture medium containing rhamnose as a carbon source. A comparable increase in naringinase activity was observed when glucose (0.124 µmol × min^−1^ × mL^−1^) was present in the culture medium. The analysis of variance did not show a statistically significant difference (significance level α = 0.05) between the naringinase activity obtained in the cultures on glucose and rhamnose. Despite this, rhamnose was selected for further research. It was taken into account because of the results of other authors who believe that *A. niger* naringinase biosynthesis is inhibited by the presence of glucose in the medium [27,29,34]. The lack of differences in the influence of rhamnose and glucose on naringinase biosynthesis by the *A. niger* KMS strain could result from the influence of metal ions on this process, especially calcium and magnesium. Gonzalez-Vazquez et al. [22] found that calcium or magnesium ions in the medium with rhamnose adversely affect the biosynthesis of extracellular naringinase by the *A. niger* ATCC1015 strain.

Rhamnose, which was selected for further research as a carbon source, was used by other authors most often at a concentration of 3.5–10 g L^−1^ [22,29,32,34,44]. High naringinase activity was achieved by Puri et al. [29] by adding rhamnose to *A. niger* MTCC 1344 culture medium. Mateles et al. [54] also reported that rhamnose or the rhamnose glucoside increased naringinase production. The maximum activity of naringinase from *A. niger* BCC 25166 was obtained by Thammawat and Pongtanya [32] by supplementing the Czapek substrate with naringin rhamnose, a carbon source.

#### 2.1.2. Selection of the Nitrogen Source in the Culture Medium

**Figure 2:**
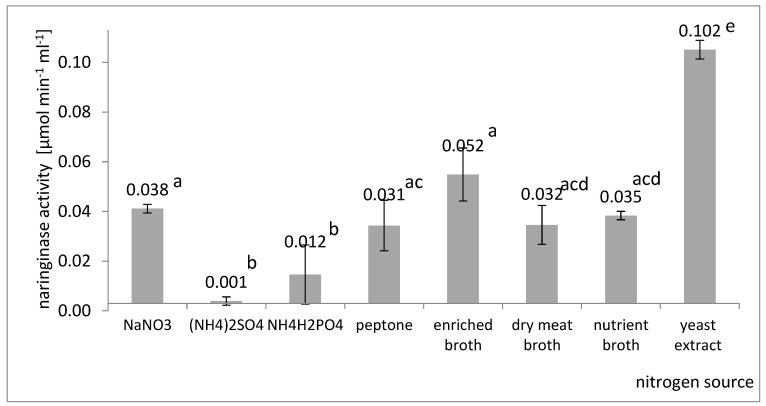
Influence of the nitrogen source on the last activity of naringinase in the post-culture fluid in submerged batch cultures with *A. niger* KMS conducted in flasks on a shaker on the Czapek medium with the addition of naringin. Various letter markings indicate statistically significant differences at *p* < 005.

Among inorganic compounds, the influence of sodium nitrate (NaNO_3_), ammonium sulfate ((NH_4_)_2_SO_4_), and ammonium dihydrogen phosphate (NH_4_H_2_PO_4_) was investigated. Among the organic compounds, the following were tested: amino acid, enriched broth, dry meat broth, plain broth, and yeast extract. The concentration of each nitrogen source was set at a certain level to ensure the same amount of total nitrogen, which was equal to 1.0 g L^−1^. A comparison of the final naringinase activities in the culture fluid obtained in submerged cultures containing the tested carbon sources is shown in Figure 2.

It was found (Figure 2) that yeast extract was the most effective in naringinase biosynthesis among all the organic nitrogen sources used. Using yeast extract as a nitrogen source allowed naringinase to obtain an activity of 0.102 µmol × min^−1^ × mL^−1^. The highest activity of naringinase in the post-culture fluid was obtained on the medium with sodium nitrate (V).

Compared to organic nitrogen sources, inorganic nitrogen sources made it possible to obtain naringinase with relatively low activity. These results are in line with the results obtained by Chen et al. [36]. It has been suggested that molds can hardly synthesize some amino acids from inorganic nitrogen sources [55]; however, Thammawat et al. [32] obtained the naringinase from *A. niger* BCC 25166 with the highest activity by using a culture medium containing NaNO_3_ as the nitrogen source. Sheheta et al. [53] found that the production of naringinase by a sea-derived *A. niger* strain can be increased by supplementing the medium in solid culture with NaNO_3_ and yeast extract.

Furthermore, other authors noticed that the addition of yeast extract, to the greatest extent, stimulates the process of naringinase production by the *Aspergillus* genus [18,23,27,41]. Studying the effect of organic and inorganic nitrogen sources on naringinase production by *Aspergillus* molds, Chen et al. [36] found that adding organic ingredients was more effective in the biosynthesis of this enzyme. Referring to the results of studies by other authors and taking into account that NaNO_3_ is a component of the Czapek substrate, and is much cheaper than yeast extract, the optimized composition of the substrate took into account the presence of both yeast extract and NaNO_3_.

#### 2.1.3. Selection of Ingredients Stimulating Naringinase Biosynthesis

**Figure 3:**
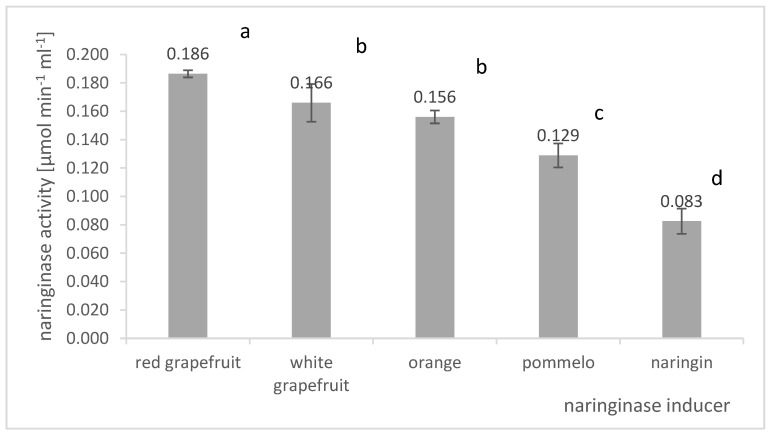
Influence of the biosynthesis stimulator on the final naringinase activity in the post-culture fluid in submerged batch cultures with *A. niger* KMS carried out in flasks on a shaker on the Czapek medium. Various letter markings indicate statistically significant differences at *p* < 0.05.

The components of the culture medium stimulating the process of naringinase biosynthesis by *A. niger* KMS were selected. The influence of naringin and dried, powdered citrus peel on naringinase production was investigated. Among citrus fruit particles, albedo, flavedo, and segmental membranes: red grapefruit, white grapefruit, orange, and pomelo were investigated as naringinase inducers. The effect of the biosynthesis stimulator on the activity of naringinase in the post-culture fluid in batch cultures of *A. niger* KMS is shown in Figure 3.

The data analysis presented in Figure 3 shows that adding powdered albedo, flavedo, and segment membranes obtained from citrus fruits stimulates naringinase biosynthesis by *A. niger* KMS. Of all the components tested, the most effective inducer of naringinase biosynthesis by *A. niger* KMS was powdered red grapefruit albedo (AFRG). We used a natural source of naringin in the form of red grapefruit albedo as an inducer of naringinase biosynthesis, which allowed us to obtain an enzyme with the activity of 0.186 µmol × min^−1^ × mL^−1^. A slightly lower activity of naringinase was also obtained when the albedo of white grapefruit (0.166 µmol × min^−1^ × mL^−1^) or orange (0.156 µmol × min^−1^ × mL^−1^) was present in the culture medium.

The team of Mendoza-Cal [33] compared the activity of the obtained naringinase in a medium containing grapefruit and orange peels. The researchers observed a higher enzyme activity in the culture medium in which grapefruit particles were present. According to the authors, it was associated with a high content of naringin in grapefruit peel (572 μmol of naringin per gram of fresh weight). Moreover, the concentration of naringinase inhibitors (fructose and glucose) was low compared to orange peel [33]. Using powdered albedo, flavedo, and red grapefruit segment membranes can allow higher naringinase activity than using naringin only. It is not only the naringin content of the powdered grapefruit skins that is crucial for naringinase production. Grapefruit powder allows high enzymatic activity in the post-culture fluid to be obtained, probably because it contains naringin, rhamnose, and other nutrients such as amino acids, proteins, and vitamins [20].

Based on the presented results, it was found that the composition of the culture medium should include albedo, flavedo, and segmental membranes of red grapefruit due to the highest naringinase activity in the post-culture fluid. In further studies, the composition of the Czapek medium was also supplemented with naringin as a distinguishing factor for inducing naringinase synthesis.

Many authors believe naringin is the best stimulator of naringinase biosynthesis [19,34]. The same conclusions were reached by Elinbaum et al. [50], who used naringin as a stimulator of the enzyme biosynthesis in solid fermentation to produce naringinase using *A. terreus*. As Bram et al. [27] reported, using a medium with naringin as an inducer increased the biosynthesis’s efficiency. Furthermore, due to their high naringin content, citrus peels are natural inducers of naringinase biosynthesis [16,20,33].

Pomelo peel powder stimulated the production of naringinase in *A. oryzae* JMU316 culture. The dried waste from pomelo juice production contains naringin, rhamnose, and other nutrients such as amino acids, proteins, and vitamins, which contribute to the high activity of naringinase in the culture medium [20].

The production of bacterial naringinase was increased by adding dried orange waste, i.e., peel, membranes, and vesicles, that were produced in the citrus processing industry to the substrate [16,20]. Puri et al. [16] observed the highest naringinase activity with a 2% content of orange powder in the culture medium. In this case, naringenin or rhamnose did not stimulate the production of naringinase; the enzyme activity was low.

#### 2.1.4. Selection of the Culture Temperature

**Figure 4:**
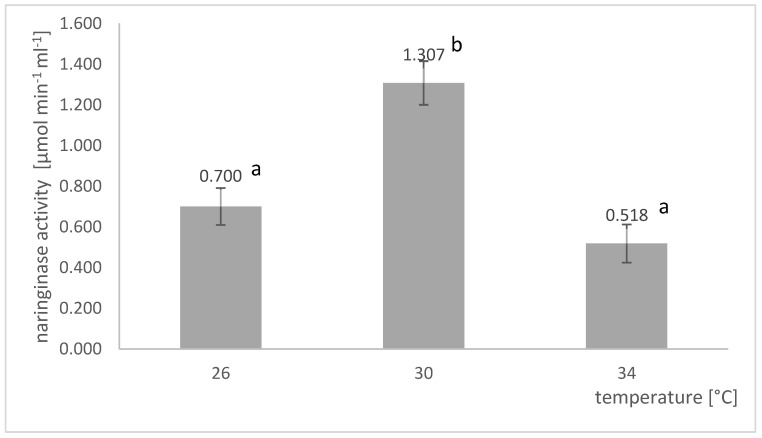
Influence of temperature on the activity of naringinase in post-culture fluid in submerged batch cultures of *A. niger* KMS conducted in shaker flasks on the Czapek medium with naringin, yeast extract, albedo, flavedo, red grapefruit segment membranes, and rhamnose. Various letter markings indicate statistically significant differences at *p* < 0.05.

A comparison of the final naringinase activities in the post-culture fluid obtained in submerged cultures conducted at different temperatures is shown in Figure 4. The temperature of *A. niger* KMS cultures grown in shaker flasks significantly impacts the naringinase activity in the post-culture fluid. The highest activity of the tested enzyme, amounting to 1.307 µmol × min^−1^ × mL^−1^, was obtained at the temperature of 30 °C.

The optimal temperature for naringinase biosynthesis by the *A. niger* KMS strain was 30 °C, which is similar to those reported in the studies of other authors. Puri et al. [29] reported that the culture temperature between 27 and 30 °C had little effect on *A. niger* MTCC1433 naringinase activity, while the enzyme that had a higher temperature (37 °C) activity was significantly reduced. Kumar et al. [34] reported that 28 °C is the optimal temperature for naringinase production from *A. niger* VB07. Borkar et al. [44] calculated that the culture of *A. niger* van Tieghem MTCC 2425 at 29.8 °C, pH 4.7, and an inducer concentration of 14.9 g L^−1^ would provide the highest naringinase activity.

### 2.2. Optimization of the Composition of the Growing Medium

The results of preliminary studies showed that the most significant influence on the synthesis of naringinase by the *A. niger* KMS strain might have five components of the medium: NaNO_3_, yeast extract, rhamnose, red grapefruit peel, segment membranes, and naringin. Potassium dihydrogen phosphate (KH_2_PO_4_) was used to assess its influence on the course of naringinase biosynthesis.

Optimized by the Box–Wilson factor–gradient method, the concentration of the six medium components in shake cultures were: NaNO_3_ (×1), yeast extract (×2), KH_2_PO_4_ (×3), albedo, flavedo, and red grapefruit segment membranes (×4), naringin (×5), and rhamnose (×6) (Table 1).

As a result of the experiments based on the Box and Wilson factor plan, the naringinase activity in the post-culture fluid obtained in the individual cultivation variants varied from 0.062 to 0.9 µmol × min^−1^ × mL^−1^. The highest activity of naringinase, equal to 0.9 µmol × min^−1^ × mL^−3^, was obtained in the cultivation variant carried out in a culture medium with the following composition (in g·100 mL^−1^): 1.28—NaNO_3_; 2.5—yeast extract; 0.257—KH_2_PO_4_; 0.8—red grapefruit peel; 0.16—naringin; 0.5—rhamnose.

The data from factor optimization were used to determine the values of the regression coefficients, which define the linear influence of the tested concentrations of the medium components on the culture results. Based on the obtained slope regression coefficients for individual factors, the jump sizes in the gradient experiment were determined for each. For the main factors, increases in the value of 0.5 g 100 mL^−1^ were assumed. The calculated increments for the less significant factors were doubled. Due to the poor solubility of naringin in aqueous solutions, an increase of 10 mg 100 mL^−1^ was assumed.

Subsequently, the factorial experiment was performed again with new levels of all six optimized substrate components. This procedure continued until the regression function’s local maximum was achieved (considering the limitations of the examined factors).

As a result of implementing the optimization plan using the gradient method, higher naringinase activities were obtained in the post-culture fluid. The naringinase activity in the culture fluid obtained in individual cultivation variants varied from 0.701 to 1.965 µmol × min^−1^ × mL^−1^. The highest activity of naringinase, equal to 1.965 µmol × min^−1^ × mL^−1^, was obtained in the culture medium with the following composition (in g·100 mL^−1^): 3.28—NaNO_3_; 3.35—yeast extract; 0.182—KH_2_PO_4_; 0.80—red grapefruit albedo; 0.17—naringin; 2.75—rhamnose. This variant was defined as a new central point used to develop a five-valued composition plan with seven repetitions in the central point (Table 2 and Table 3).

The data analysis presented in Table 3 showed that the highest activity of naringinase obtained in individual cultivation variants varied from 0.993 to 2.210 µmol × min^−1^ × mL^−1^. The highest activity of naringinase, (2.210 µmol × min^−1^ × mL^−1^) was obtained as a result of cultivation in a medium with the following composition (in g·100 mL^−1^): 3.78—NaNO_3_; 3.62—yeast extract; 0.187—KH_2_PO_4_; 0.86—albedo, the flavedo, and red grapefruit segmental membranes; 0.185—naringin; 3.25—rhamnose.

The results obtained in this stage of the study were subjected to the ANOVA statistical analysis of variance to determine the significant effects and the values of the coefficients of the quadratic regression model using the response surface of the determined model as described. After the reduction of the most significant factors, the test results presented in Table 3 can be described by the following quadratic regression model of the response surface:

(1) $$f=2.1791-0.0962x_{1\;}^{2}-0.0772x_{2}^{2}+0.0504x_{3}-0.0889x_{3}^{2}+0.0635x_{4}-0.0546x_{4}^{2}-0.0455x_{5}-0.1248x_{5}^{2}+0.0334x_{6}-0.0744x_{6}^{2}+0.0707x_{1}x_{2}+0.0396x_{1}x_{3}+0.0889x_{1}x_{5}-0.0551x_{1}x_{6}-0.0507x_{2}x_{3}+0.0640x_{2}x_{4}+0.0423x_{3}x_{4}-0.0381x_{3}x_{6}$$

The determined equation describing the response surface was used to determine the predictable activity of naringinase in the analyzed area of the variability of the concentration of substrate components. It was also the basis for determining, using the MATLAB program, the optimal composition of the medium, thereby allowing for the maximum activity of naringinase in the post-culture fluid. The conducted research allowed us to determine the optimal concentrations (g 100 mL^−1^) of the following factors: 3.3317—NaNO_3_; 3.4274—yeast extract; 0.1841—KH_2_PO_4_; 0.8548—red grapefruit albedo; 0.1678—naringin; 2.7891—rhamnose. The cultivation of the *A. niger* KMS strain on such a medium allows for obtaining naringinase with an activity of 2.2235 µmol × min^−1^ × mL^−1^.

The obtained results made it possible to redefine the composition of the culture medium to obtain the highest naringinase activity in the culture fluid. Kumar [19], in research on the influence of various inducers, proved that the best stimulator of naringinase synthesis by *A. niger* MTCC 1344 is naringin at a concentration of 0.1%. Similar results were obtained for the production of naringinase from *A. niger* VB07 [34]. The optimal concentration of naringin was 0.1%, and the highest activity of naringinase was observed after seven days of its biosynthesis.

Citrus particles in the processes of naringinase preparation described in the literature were used at a concentration from 1.47 to 2.7% [16,20,33,44,51]. The optimal yeast extract concentration for naringinase biosynthesis by *A. niger* KMS was 3.363 g 100 mL^−1^. It was a much lower value than that used in the research for optimizing the composition of the culture medium for naringinase biosynthesis by the same strain on a glucose-containing medium [43]. Furthermore, Ni et al. [23] used a slight 0.1% yeast extract concentration. It turned out that the concentration of yeast extract equal to 3.636 g 100 mL^−1^ and sodium nitrate equal to 4.011 g 100 mL^−1^ allowed for the highest naringinase activity in the post-culture fluid of *A. niger* KMS strain to be obtained.

Other nitrogen sources such as urea and diammonium hydrogen phosphate, according to Puri et al. [29], inhibit naringinase production by *A. niger;* this was also confirmed by the results of studies on naringinase biosynthesis by *A. niger* KMS strain.

**Figure 5:**
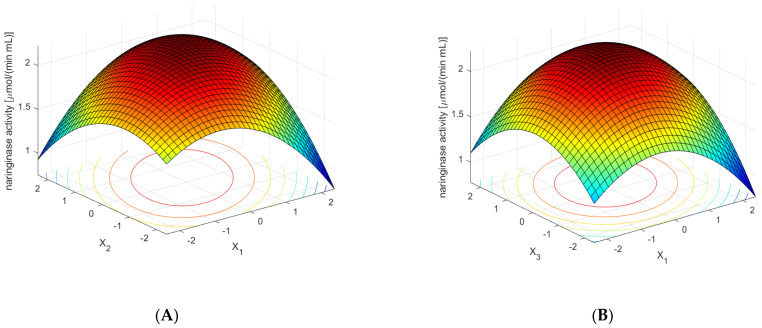

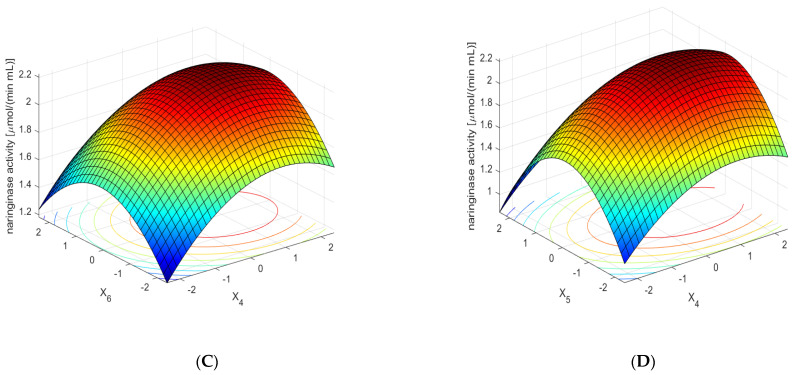
Effect of the concentrations of sodium nitrate (×1), yeast extract (×2), potassium dihydrogen phosphate (×3), red grapefruit albedo (×4), naringin (×5), and rhamnose (×6) on the activity of naringinase in *A. niger* KMS post-culture medium. The concentration of the substrate components was expressed in dimensionless values. Each figure shows the dependence of the activity on the two components of the medium at optimal concentrations of the remaining ones. (**A**)—Effect of the concentrations of sodium nitrate (×1) and yeast extract (×2) on the activity of naringinase in *A. niger* KMS post-culture medium. (**B**)—Effect of the concentrations of sodium nitrate (×1) and potassium dihydrogen phosphate (×3) on the activity of naringinase in *A. niger* KMS post-culture medium. (**C**)—Effect of the concentrations of red grapefruit albedo (×4), and rhamnose (×6) on the activity of naringinase in *A. niger* KMS post-culture medium. (**D**)—Effect of the concentrations of red grapefruit albedo (×4) and naringin (×5) on the activity of naringinase in *A. niger* KMS post-culture medium.

Figure 5A shows the dependence of naringinase activity in the post-culture fluid on the concentration of NaNO_3_ and yeast extract—along with the optimal values of the remaining components of the medium. The presented relationship shows that the highest activity of naringinase was obtained in a medium that is relatively rich in yeast extract and NaNO_3_. The low content of one of the components and the high concentration of the other simultaneously reduce the activity of naringinase in the post-culture fluid.

Figure 5B shows the influence of KH_2_PO_4_ and NaNO_3_ concentrations on the activity of naringinase. The other variables were at the optimal value. High naringinase activity was obtained at KH_2_PO_4_ and NaNO_3_ concentrations.

Figure 5C shows the effect of the concentration of albedo, flavedo, red grapefruit segment membranes, and rhamnose on the activity of naringinase in the post-culture fluid, with the optimal composition of the remaining medium components. The dependence presented in the figure shows that the high content of powdered albedo in the entire analyzed range of rhamnose concentration induces naringinase biosynthesis to the greatest extent.

Figure 5D shows the effect of naringin concentration and red grapefruit albedo on the activity of the tested enzyme in the cultivated liquid—along with the values of the remaining components of the medium constant. The dependence presented in the figure shows that obtaining the maximum activity of naringinase using a high concentration of powdered albedo and grapefruit flavedo is possible only with the optimal content of naringin.

### 2.3. Receiving a Naringinase Preparation

#### 2.3.1. Obtaining a Solid Naringinase Preparation

As a result of cultivating *A. niger* KMS in optimal conditions, a post-culture fluid with the activity of 2.111 ± 0.121 µmol × min^−1^ × mL^−1^ was maintained. After concentrating the culture fluid, the naringinase activity was 40.56 ± 0.89 µmol × min^−1^ × mL^−1^. The authors of many studies often do not provide information on whether the naringinase activity was determined in the non-concentrated or concentrated post-culture fluid, which makes it difficult to compare the test results. After precipitation of the protein with cooled acetone, the *A. niger* KMS-derived naringinase preparation was obtained with an activity of 816.0 ± 10.6 µmol × min^−1^ × mL^−1^. Table 4 shows the results of the purification of the naringinase preparation. 

Naringinase from *A. niger* DB056 with similar activity in the concentrated post-culture fluid (36.7 µmol × min^−1^ × mL^−1^) was obtained by Ni et al. [23]. The precipitated naringinase preparation, however, had a lower activity (37.6 µmol × min^−1^ × mL^−1^).

Kumar et al. [34] received naringinase with a higher activity in *A. niger* VB07 culture fluid, which amounted to 17.28 µmol × min^−1^ × mL^−1^. The optimized culture medium contained naringin (0.1%), rhamnose (0.5%), peptone (0.25%), and glycine (10 mM). In another study, Kumar [19] obtained naringinase from *A. niger* MTCC 344 with an activity of 9.68 µmol × min^−1^ × mL^−1^ on a medium containing naringin (0.1%), molasses (1.5%), peptone (0.5%), and mineral salts; however, it is not easy to compare the results of these actors because they measured naringinase activity at a higher temperature (60 °C).

Some authors obtained significantly higher naringinase activity in the culture fluid of various *A. niger* strains. Igbonekwu et al. [35] obtained a culture fluid with an *A. niger* naringinase activity equal to 157.7 µmol × min^−1^ × mL^−1^. After thorough purification, they obtained a naringinase activity of 917.2 µmol×min^−1^ × mL^−1^. However, the activity results may raise doubts because the authors reported that they used a 5% naringin solution to determine the activity of naringinase. Nevertheless, the solubility of this compound is much lower (it is 1 mg per 1 mL at 40 °C).

The maximum specific activity of naringinase from *A. niger* BCC 25166, amounting to the activity of 117.77 µmol × min^−1^ × mg^−1^ of protein, was obtained on the medium containing rhamnose (0.375%), naringin (0.1%), sodium nitrate (0.25%), and other components of the substrate Czapek [32]; however, it is difficult to compare the specific activity obtained in the culture of the *A. niger* KMS strain with these results because the composition of the medium contained yeast extract, which resulted in a high protein content in the post-culture fluid.

In turn, as a result of culturing *A. niger* van Tieghem MTCC 2425 under optimized conditions on a medium containing orange particles, the activity of the naringinase preparation was about 550 µmol × min^−1^ × g^−1^ [44]. The enzyme preparation was obtained by concentration by ultrafiltration and ammonium sulfate precipitation. The *A. niger* KMS naringinase preparation was obtained using similar operations and its activity was over 800 U g^−1^.

The authors who used other species and types of microorganisms obtained a relatively low activity of naringinase. The cultivation of the *A. oryzae* JMU316 strain on a medium containing pomelo particles, peptone, and mineral salts allowed them to obtain naringinase with an activity of 0.7 µmol × min^−1^ × mL^−1^ [20]. As a result of culturing the *A. aculeatus* JMUdb058 strain on an optimized medium containing, among other things, yeast extract, naringin, and soy flour, a naringinase activity of 1.16 µmol × min^−1^ × mL^−1^ was obtained [36].

#### 2.3.2. Protein Separation of the Naringinase Preparation by Size Exclusion Chromatography

**Figure 6:**
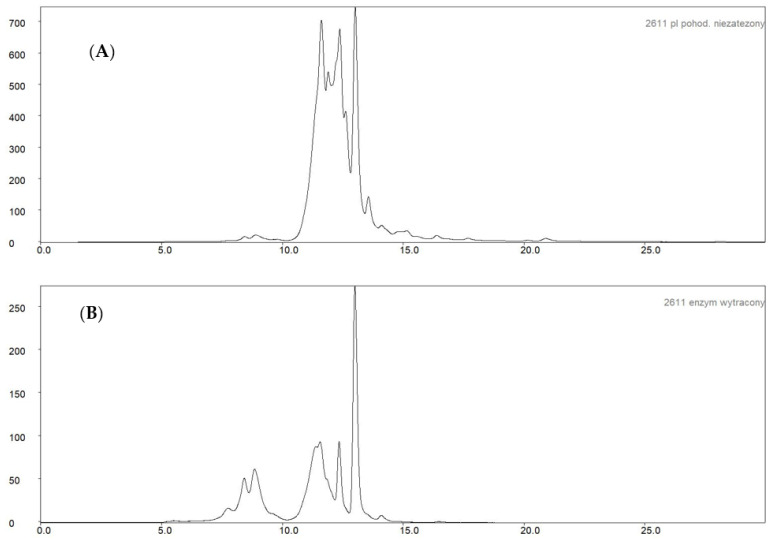
Chromatogram of size exclusion chromatography: (**A**)—*A. niger* KMS post-culture fluid; (**B**)—the obtained naringinase preparation.

In order to illustrate the effectiveness of the post-culture fluid purification, the distribution of proteins differing in molecular weight was examined using gel permeation chromatography on a YarraTM 3 μm SEC-3000 column. The post-culture fluid and the precipitated enzyme preparation were tested. The results, in the form of chromatograms, are shown in Figure 6. As a result of the purification of the culture fluid and preparation of the naringinase preparation, significant removal of low molecular weight proteins—less than 50 kDa—with a retention time greater than 10 min was observed. An increase in the proportion of proteins with high molecular weight concerning low molecular weight proteins was observed.

## 3. Conclusions

Using waste in the form of grapefruit skins as components of the culture medium for the biosynthesis of naringinase, which can remove the bitter taste of grapefruit juices, is an effective method of managing this citrus fruit. Developing a method of naringinase biosynthesis on a substrate containing natural stimulators of the production of this enzyme, derived from waste materials or by-products from grapefruit juice, e.g., citrus peels, will enable their better management. These natural citrus fruit fragments are a rich source of carbon and substances that stimulate naringinase biosynthesis. The Box–Wilson method was confirmed to optimize naringinase biosynthesis by *A. niger* KMS in shake-flask cultures. Environmental factors (pH and dissolved oxygen) can also influence the production of naringinase by the *A. niger* KMS. Further studies on naringinase biosynthesis should be carried out in a bioreactor.

## 4. Materials and Methods

### 4.1. Materials

#### 4.1.1. Microorganisms

*A. niger* KMS strain was used in the research, selected from among six *A. niger* strains (KMS, PSR, X/IX, C-WL, LX, LP) from the microbial collection of the Department of Biotechnology and Food Analysis of the Institute of Food Chemistry and Technology of the University of Economics in Wrocław [43].

#### 4.1.2. Culture Media

The following substances were used to prepare the culture media: MgSO_4_ · 7 × H_2_O, p.a., KH_2_PO_4_, part a.a., FeSO_4_ 7H_2_O p.a., KCl, p.a. (POCH S.A. Gliwice) NaNO_3,_ p.a. (Eurochem BGD Sp. z o.o. Tarnów, Poland), -naringin (naringenin-7-rhamnosidoglucoside) from citrus fruit with a purity above 90%, (Sigma-Aldrich, Poznań, Poland), yeast extract (BTL Sp. z o.o. Department of Enzymes and Peptides, Łódź, Poland); L-rhamnose, 99% pure (Sigma-Aldrich, Poznań, Poland). Albedo, flavedo, and segment membranes were obtained from Star Ruby red grapefruit from Turkey. The obtained fruit particles were dried at 30 °C and ground to a powder.

#### 4.1.3. Substrates to Choose the Carbon Source

When selecting the carbon source in the culture medium, the following saccharides were used: rhamnose, glucose, sucrose, starch, maltose, or raw materials or by-products of processing processes, such as glycerol and molasses. In a concentration of 10 g L^−1^, carbon sources were added to the medium of Czapek with naringin (0.5 g L^−1^). The substrates were supplemented with water.

#### 4.1.4. The Medium for Selecting Ingredients Stimulating the Naringinase Biosynthesis

Naringin and dried powdered albedo, flavedo, and segment membranes of red grapefruit, white grapefruit, orange, and pomelo were used to select ingredients stimulating naringinase biosynthesis in the culture medium. The concentration of components inducing naringinase production was 10 g L^−1^. The Czapek medium supplemented with the inducer of naringinase biosynthesis was used at this research stage. The substrates were supplemented with water. 

#### 4.1.5. The Medium for Selecting the Temperature of *A. niger* Culture

The research on the selection of the temperature of *A. niger* mold cultivation was carried out with the use of the Czapek medium supplemented with (in g·L^−1^): sodium nitrate (V)—7.8; yeast extract—20.0; potassium dihydrogen phosphate (V)—1.57, albedo, the flavedo, and red grapefruit segment membranes—5.0; naringin—1.2; rhamnose—2.5. The substrate was replenished with water.

#### 4.1.6. The Medium for Optimization

In the research on the optimization of the concentration of the components of the culture medium, the following were used: sodium nitrate in the range from 0 to 47.8 g·L^−1^, yeast extract from 1 to 47.8 g·L^−1^, potassium dihydrogen phosphate 0.5 to 1. 97 g·L^−1^, red grapefruit albedo 5 to 9.8 g·L^−1^, naringin 0.1 to 2 g·L^−1^, and rhamnose from 0 to 42.5 g·L^−1^. The values of the concentrations of the individual components of the substrate resulted from the optimization plan applied using the Box and Wilson factor-gradient method. The substrates were supplemented with water.

### 4.2. Analytical Methods

#### 4.2.1. Determination of Naringinase Activity in the Culture Fluid

The Davis colorimetric method determined naringinase activity in the culture fluid [56]. Sample of the culture fluid was centrifuged and, if necessary, diluted with distilled water. A total of 0.2 mL of the culture fluid was combined with 0.3 mL of 0.1 M McIlvaine buffer pH 4.0 and 1 cc of 0.1% naringin solution. Everything was incubated in a thermostat at 50 °C for 30 min. The absorbance of the solutions was measured at a wavelength of λ = 420 nm using a Marcel MEDIA spectrophotometer (Marcel Sp.z o.o., Warsaw, Poland). The absorbance was then converted into naringin concentration using the regression equation characterizing the standard curve, prepared based on measuring the absorbance of aqueous naringin solutions in the concentration range of 50–1000 µg cm^−3^. The naringinase activity is expressed in μmoles of naringin hydrolyzed per 1 min by 1 mL of culture medium.

#### 4.2.2. Determination of the Activity of the Naringinase Enzyme Preparation

A total of 5 mg of the naringinase preparation was dissolved in 10 mL of 0.9% sodium chloride. A total of 0.2 mL of the enzyme solution was combined with 0.3 mL of 0.1 M acetate buffer pH 4.0 and 1 mL of 0.1% naringin solution. Everything was incubated in a thermostat at 50 °C for 30 min. The Davis method [56] was used to determine the concentration of naringin in the reaction mixture. The naringinase activity is expressed in μmoles of naringin hydrolyzed per 1 min by 1 g of the enzyme preparation.

#### 4.2.3. Protein Separation of the Naringinase Preparation by Size Exclusion Chromatography

Proteins were separated by size exclusion chromatography on a YarraTM 3 µm SEC-3000 column (300 × 7.8 mm). The analysis was performed using a Parkin Elmer high-pressure chromatograph with UV-Vis and RI detectors. The following separation conditions were used: phase 0.1 M phosphate buffer, pH 6.8; 0.025% NaN_3_; flow rate 1 mL·min^−1^; wavelength 280 nm. The LP-Chrom ver.1.54 software was used for the analysis of the chromatograms.

### 4.3. Culture of A. niger KMS Strain

Prepared, sterile media were inoculated by adding 0.2 mL of *A. niger* spore suspension (3 × 107 spores in 1 cm^3^ of water). The cultures were carried out in 500 mL Duran–Schott septum flasks with 100 mL of medium each. The flasks were placed on a GLF 3031 shaker with a frequency of 150 min^−1^ movements for seven days. During the shaking flasks’ cultivation of agitated molds of *A. niger*, 2 mL of samples were collected every 24 h to determine the activity of naringinase in the post-culture fluid. At the end of the culture, no increase in naringinase activity in the culture fluid was assumed.

### 4.4. Optimization of the Composition of the Growing Medium

The Box and Wilson factorial gradient method was used to optimize the culture medium composition. The concentrations of sodium nitrate, yeast extract, potassium dihydrogen phosphate (V), albedo, and flavedo of red grapefruit, naringin, and rhamnose were considered. In factor optimization, the values of the lower (−1) and upper (1) levels of the optimized parameter were determined for each of the six given culture medium components. Based on the results of factor optimization, linear regression coefficients were calculated, which were used to determine the direction and magnitude of the change in concentration of each of the optimized components (gradient optimization). The obtained results of the gradient optimization were used to determine a new central point and prepare a composition plan. Based on the results of the conducted experiments, the regression equation describing the response surface was determined, taking into account only statistically significant coefficients. The obtained function was used to calculate the maximum naringinase activity in the analyzed area of the variability of substrate components concentrations. The optimal point was determined using the hybrid method (combination of the genetic algorithm and the classical method) using the MATLAB optimization package.

## Figures and Tables

**Table 1:** List of substrate components subjected to optimization.

Parameter Number	Component	Component Concertation [g 100 mL^−1^]			
		−1 *	1 *	0	Δ
x_1_	NaNO_3_	0.28	1.28	0.78	0.5
x_2_	yeast extract	1.5	2.5	2.0	0.5
x_3_	KH_2_PO_4_	0.057	0.257	0.157	0.1
x_4_	dry albedo, flavedo, and red grapefruit segment membranes	0.2	0.8	0.5	0.3
x_5_	naringin	0.08	0.16	0.12	0.04
x_6_	rhamnose	0.0	0.5	0.25	0.25

* (−1—lower, 1-upper concentration level of the factor (dimensionless values).

**Table 2:** Concentrations of the substrate components subjected to gradient optimization.

Parameter Number	Component	Component Concertation [g·100 mL^−l^]					
		−2.38	2.38	−1 *	1 *	0	Δ
x_1_	NaNO_3_	2.09	4.47	2.78	3.78	3.280	0.5
x_2_	yeast extract	2.71	3.99	3.08	3.62	3.350	0.27
x_3_	KH_2_PO_4_	0.170	0.19	0.177	0.187	0.182	0.005
x_4_	dry albedo, flavedo, and red grapefruit segment membranes	0.66	0.94	0.74	0.86	0.800	0.06
x_5_	naringin	0.134	0.205	0.155	0.185	0.170	0.015
x_6_	rhamnose	1.56	3.94	2.25	3.25	2.750	0.5

* (−1—lower, 1-upper concentration level of the factor (dimensionless values).

**Table 3:** Concentrations of optimized components and experimental results according to the Box–Wilson central composition plan.

Culture Number	Component Concertation [g·100 mL^−1^]						Naringinase Activity [µmol × min^−1^ × mL^−1^]
	x_1_	x_2_	x_3_	x_4_	x_5_	x_6_	
**1**	−1	−1	−1	−1	−1	−1	1.560
**2**	−1	−1	−1	−1	1	1	1.732
**3**	−1	−1	−1	1	−1	1	2.110
**4**	−1	−1	−1	1	1	−1	1.269
**5**	−1	−1	1	−1	−1	1	1.785
**6**	−1	−1	1	−1	1	−1	1.350
**7**	−1	−1	1	1	−1	−1	1.764
**8**	−1	−1	1	1	1	1	1.850
**9**	−1	1	−1	−1	−1	1	1.876
**10**	−1	1	−1	−1	1	−1	1.162
**11**	−1	1	−1	1	−1	−1	1.641
**12**	−1	1	−1	1	1	1	1.801
**13**	−1	1	1	−1	−1	−1	1.786
**14**	−1	1	1	−1	1	1	0.993
**15**	−1	1	1	1	−1	1	1.758
**16**	−1	1	1	1	1	−1	1.870
**17**	1	−1	−1	−1	−1	1	1.269
**18**	1	−1	−1	−1	1	−1	1.649
**19**	1	−1	−1	1	−1	−1	1.600
**20**	1	−1	−1	1	1	1	1.188
**21**	1	−1	1	−1	−1	−1	1.784
**22**	1	−1	1	−1	1	1	1.839
**23**	1	−1	1	1	−1	1	1.601
**24**	1	−1	1	1	1	−1	1.854
**25**	1	1	−1	−1	−1	−1	1.700
**26**	1	1	−1	−1	1	1	1.827
**27**	1	1	−1	1	−1	1	1.903
**28**	1	1	−1	1	1	−1	1.878
**29**	1	1	1	−1	−1	1	1.699
**30**	1	1	1	−1	1	−1	1.500
**31**	1	1	1	1	−1	−1	1.798
**32**	1	1	1	1	1	1	2.210
**33**	−2.38	0	0	0	0	0	1.800
**34**	2.38	0	0	0	0	0	1.400
**35**	0	−2.38	0	0	0	0	1.699
**36**	0	2.38	0	0	0	0	1.716
**37**	0	0	−2.38	0	0	0	1.450
**38**	0	0	2.38	0	0	0	1.832
**39**	0	0	0	−2.38	0	0	1.800
**40**	0	0	0	2.38	0	0	1.871
**41**	0	0	0	0	−2.38	0	1.504
**42**	0	0	0	0	2.38	0	1.374
**43**	0	0	0	0	0	−2.38	1.687
**44**	0	0	0	0	0	2.38	1.760
**45**	0	0	0	0	0	0	2.200
**46**	0	0	0	0	0	0	2.167
**47**	0	0	0	0	0	0	2.200
**48**	0	0	0	0	0	0	2.190
**49**	0	0	0	0	0	0	2.200
**50**	0	0	0	0	0	0	2.110
**51**	0	0	0	0	0	0	2.210

(−1—lower, 1-upper concentration level of the factor (dimensionless values).

**Table 4:** Results of obtaining a naringinase preparation from an *A. niger* KMS culture.

Obtaining Stage	Activity [μmol· min^−1^·mL^−1^]	Volume or Quantity [ml]	Total Activity [μmol· min^−1^]	Process Efficiency [%]	Specific Activity [μmol·min^−1^ mg^−1^ Protein]	Protein Concentration [mg·ml^−1^]
**Post-culture fluid**	2.11	1600	3376	100	0.208	10.14
**The fluid** **after** **concentration by** **ultrafiltration**	40.56	70.2	2847.3	84.3	1.357	29.88
**Precipitation with cold** **acetone**	816 μmol·min^−1^· g^−1^ *	2.9 g *	2366.4	70.1	1.563	522 mg·g^−1^ *

*—solid preparation.

## Data Availability

Data related to this study is available from the corresponding author upon reasonable request.
